# Protective effects of hydrogen gas in a rat model of branch retinal vein occlusion via decreasing VEGF-α expression

**DOI:** 10.1186/s12886-019-1105-2

**Published:** 2019-05-16

**Authors:** Pan Long, Weiming Yan, Mengshan He, Qianli Zhang, Zhe Wang, Manhong Li, Junhui Xue, Tao Chen, Jing An, Zuoming Zhang

**Affiliations:** 1 Center of Clinical Aerospace Medicine, Fourth Military Medical University, No.169 Changle West Road, Xi’an, 710032 Shaanxi China; 2 Department of Ophthalmology, The 900th Hospital of the Joint Logistics Team of Chinese PLA, Fuzhou, 350025 Fujian China; 3 Department of Chinese Material Medical and Natural Medicines, Fourth Military Medical University, Xi’an, 710032 Shaanxi China; 4 Company 11 Brigade 4, College of Basic Medicine, Fourth Military Medical University, Xi’an, 710032 Shaanxi China; 5 Department of Ophthalmology of Xijing Hospital, Fourth Military Medical University, Xi’an, 710032 Shaanxi China; 6 Institute of Neurobiology, School of Basic Medical Sciences, Xi’an Jiaotong University, No.76 Yanta Weast Road, Xi’an, 710061 Shaanxi China

**Keywords:** Hydrogen gas, Branch retinal vein occlusion, Electroretinography, Vascular endothelial growth factor

## Abstract

**Background:**

Oxidative stress (OS) is an essential factor in the pathogenesis of branch retinal vein occlusion (BRVO). Studies have demonstrated the role of hydrogen gas in the regulation of OS. This study was designed to evaluate the efficacy of hydrogen gas on the BRVO rat model.

**Methods:**

Twenty-four BRVO rats were randomly divided into two groups: the hydrogen gas (H) group (42% H_2_, 21% O_2_, 37% N_2_) and the model (M) group (21% O_2_, 79% N_2_). Rats in the H group inhaled hydrogen gas for 8 h every day up to 30 d post-occlusion. Twelve age-matched healthy rats served as the control (C) group. Retinal function and morphology were detected at 1, 7, 14 and 30 d post-occlusion. Furthermore, the expression of vascular endothelial growth factor (VEGF-α) was detected by immunofluorescent staining.

**Results:**

Full-field electroretinography (ffERG) revealed that the amplitude of the b-wave (dark-adaptation 3.0 response), the amplitude of the OPs2 wave and the light-adapted flicker response in the H group were all higher than those in the M group at 7 d post-occlusion (all *p <* 0.05). The reopen time of occlusive retinal vessels in the H group was 2.235 ± 1.128 d, which was shorter than that in the M group (4.234 ± 2.236 d, *p* < 0.05). The rats in the H group had a thinner IPL + GCL + NFL and an increased total retina compared with those in the M group at 3 d post-occlusion (*p* < 0.05), while the rats in the H group had a thicker INL, IPL + GCL + NFL and total retina compared with those at 7, 14 and 30 d post-occlusion (*p* < 0.05). Moreover, the flow velocity of ear vein blood was increased in the H group compared with that in the M group (*p* < 0.05). The expression of VEGF-α in the H group was dramatically decreased compared with that in the M group at 1, 7 and 14 d post-occlusion (*p* < 0.05), while the expression kept in similar level at 30 d post-occlusion (*p > 0.05*).

**Conclusions:**

Our findings demonstrate that inhalation of hydrogen gas could alleviate retinal oedema, shorten reopen time and improve retinal function, and the potential mechanism might be related to a decrease in VEGF-α expression.

## Background

Retinal vein occlusion (RVO) is mainly classified with central retinal vein occlusion (CRVO) and branch retinal vein occlusion (BRVO) according to the occlusive vein sites. It is well known that a severe sight-threatening ophthalmic vessel disease most commonly affects middle-aged ranging to older people [1, 2]. Compared with CRVO, BRVO features higher morbidity and a larger therapeutic window of opportunity, which should be optimized with management [3]. Currently, first-line therapeutic methods include glucocorticoid, anti-VEGF, laser photocoagulation, and traditional herb medicines (mostly in eastern countries) [4]. However, those treatment methods cannot meet the needs of some individuals. Specifically, glucocorticoid is convenient but inevitably produces various adverse side effects, such as increased susceptibility to infection, damage to the optic nerve, cataracts, and so on [5–7]. Although adverse events following anti-VEGF therapy are rare, it would be relevant to address these challenges, including frequent visits for injections [8], recurrent macular oedema [9, 10], anti-VEGF non-responders [9], time-dependent effects [11] and high expenses related to the agents [12]. Conventional medicine could not produce satisfactory results because of the blood-retina barrier and the unique structure of the retina. Therefore, it is urgent to explore effective measures against BRVO with evident therapeutic effects and fewer adverse side effects.

Recently, hydrogen gas, as a novel inert gas, has aroused much interest in medical areas. A remarkable achievement is clinical research confirming that inhalation of hydrogen gas could alleviate brain ischaemia/reperfusion (I/R) injury via anti-inflammation and antioxidation [13]. More experimental evidence, including studies on the brain, heart, liver, lung, kidney and eye, has confirmed the beneficial role of the inhalation of hydrogen gas. Furthermore, our laboratory previously applied hydrogen gas in several ophthalmic diseases, including retina injury induced by intense light [14], retinitis pigmentosa induced by methylnitrosourea (MNU) [15], dry eyes and endotoxin-induced uveitis [16], which verified the potential value of hydrogen gas on ophthalmic diseases.

In this study, we proposed the hypothesis that hydrogen gas could be a new therapeutic method for treating BRVO, collected therapeutic evidence of hydrogen gas and explored the relationship with the expression of VEGF-α.

## Methods

### Animals

Forty-two adult male Sprague-Dawley (SD) rats (6–8 weeks, 180–220 g) were obtained from the Laboratory Animal Center of the Fourth Military Medical University (license No. 2014270138S). The rats were raised and tested under the condition with food and water free intake and maintained room temperature 23 °C ± 3 °C, humidity 45–65%, and 12 h light/12 h dark cycle. In this study, the experimental protocols were approved by the ethical committee of the Animal Care and Experimental Committee of the Fourth Military Medical University. All experiments were in accordance with the Association for Research in Vision and Ophthalmology (ARVO) statements for ophthalmic research animal use. Rats were euthanized with lethal sodium pentobarbital (3%, 10 ml/ kg) (Sigma, St Louis, MO, USA) at each detection point.

### BRVO model

Thirty rats were anaesthetized by intraperitoneal injection (IP) with 1% sodium pentobarbital (3mL/kg) and sumianxin II (50 μL each rat) (Jilin Shengda Animal Pharmaceutical Co., Ltd., Jilin, China), and 50 mg/mL Rose Bengal (Chengdu aikeda reagent co. Ltd., Sichuan, China) was injected into the rat tail vein 1 min before laser applied. Additionally, the right eye was dilated with 0.5% tropicamide (Shenyang Xingji Co., Ltd., Liaoning, China). Then, bifurcation of the retinal vein secondary vasculature was found using a Micron IV Retinal Imaging Microscope (Lumenis, Inc., USA), and 50 laser spots were applied with the set parameters (power: 80 mW, duration: 100 ms; spot size: 100 μm).

### Hydrogen gas administration

The mixed gas was produced by an AMS-H-01 hydrogen-oxygen nebulizer (Asclepius Medites Co., Shanghai, China), which contained 67% H_2_ and 33% O_2_ from water by electrolysis. Furthermore, N_2_ was applied to modulate the concentration of O_2_ at 21% equal with the conventional condition. Rats in the hydrogen gas (H) group inhaled the gas mixture (21% O_2_, 42% H_2_, 37% N_2_) at 3 L/min for 8 h (once/day, 30 d). Additionally, the concentration of hydrogen gas was monitored by thermal trace GC ultra-gas chromatography (Thermo Fisher, MA, USA) and maintained at 42% concentration throughout the study. The rats were placed into a special closed gas chamber and moved freely. Rats in the model (M) group and the control (C) group did not receive any treatment.

### Electroretinography

FfERG measurement was performed as an international electrophysiological standard (ISCEV), including dark-adapted 0.01 response, dark-adapted 3.0 response, dark-adapted oscillatory potentials, light-adapted 3.0 response and light-adapted flicker response at 1, 7, 14 and 30 d post-occlusion as previously described [17–19]. Briefly, rats were adapted in a dark environment for 12 h for dark adaptation. All operation procedures were performed in a dim, red-light room. Rats were anaesthetized as described above. The pupils were dilated with 0.5% tropicamide ahead of ERG operation. FfERG was recorded using full-field (Ganzfeld) stimulation with a computer system (RETI port; Roland Consult GmbH, Brandenburg, Germany). The recording electrode was a custom-made silver chloride electrode placed softly on the centre of the cornea. Stainless steel needle electrodes were placed in the cheek and tail to serve as the reference and ground electrodes, respectively. Gatifloxacin eye gel (Shenyang Xingji Co., Ltd., Liaoning, China) was used three times a day after ERG testing to avoid infection.

### OCT, fundus photograph and ear microcirculation detection

OCT image detection was used on 1, 7, 14 and 30 d post-occlusion. The detection procedure complied with the operator manual. The right eye had previously been dilated with 0.5% tropicamide. Then, gatifloxacin eye gel was used as coupling gel to protect the rat cornea from injury. Fundus and OCT images were captured from 20 positions for each eye using a Retinal Imaging System (OPTO-RIS, OptoProbe, Canada) and a 4D-ISOCT Microscope Imaging System (ISOCT, OptoProbe, Canada). The thickness of the retinal layers was calculated with OCT Image Analysis Software (OptoProbe, Version 2.0, Canada). Furthermore, BRVO rat model ear microcirculation was detected by a microcirculation detector (Xindi Co., Ltd., Shanghai, China).

### Immunofluorescence staining

Immunofluorescence staining was performed according to the manufacturer’s instructions at 1, 7, 14 and 30 d post-occlusion (*n* = 3). Paraffin eye sections were deparaffinized in dimethylbenzene and dehydrated in gradient ethyl alcohol. Then, the sections were washed in phosphate buffer saline (PBS; 0.1 mM, pH 7.2) 3 times for 5 min. Antigen retrieval solution (9 mL 0.1 mmol/L citric acid, 41 mL 0.1 mmol/L sodium citrate, 450 mL ddH_2_O) was performed with a medium baking temperature for 10 min. Next, the sections were washed in PBS 3 times for 5 min. Then, 10% goat serum was applied for 2 h, and the sections were incubated with anti-VEGF-α (#GTX102643, Gene Tex, Irvine, CA) at a 1:100 dilution overnight at 4 °C. Slides incubated without primary antibody served as control slides. The slides were washed with PBS 3 times for 5 min and incubated with Cy3 marked goat anti-rabbit IgG (H + L) fluorescence secondary antibody (Zhuangzhi, EK022, Xi’an, Shaanxi Province, China) at 1:200 dilution for 1 h at room temperature. Slides were again washed in PBS 3 times for 5 min. DAPI (100 ng/mL) was used to stain nuclei for 10 min. Images of the slides were captured on a fluorescence microscope (BX53, Olympus, Japan).

### Statistical analyses

Analysis of variance (ANOVA) followed by Bonferroni’s post hoc analysis was performed to examine the significant differences among all groups unless otherwise specified; the values were presented as the mean ± standard deviation (SD), with *p* ≤ 0.05 considered statistically significant.

## Results

### BRVO rat model

The BRVO model was confirmed by OCT and fundus photography. Twenty-four BRVO rats were constructed successfully, a success rate of 80% (24/30). Moreover, 24 BRVO rats were randomly divided into 2 groups: the hydrogen gas (H) group (*n* = 12) and the model (M) group (*n* = 12). Twelve age-matched male rats served as the control (C) group.

### Effects of hydrogen gas on retinal function

**Fig. 1:**
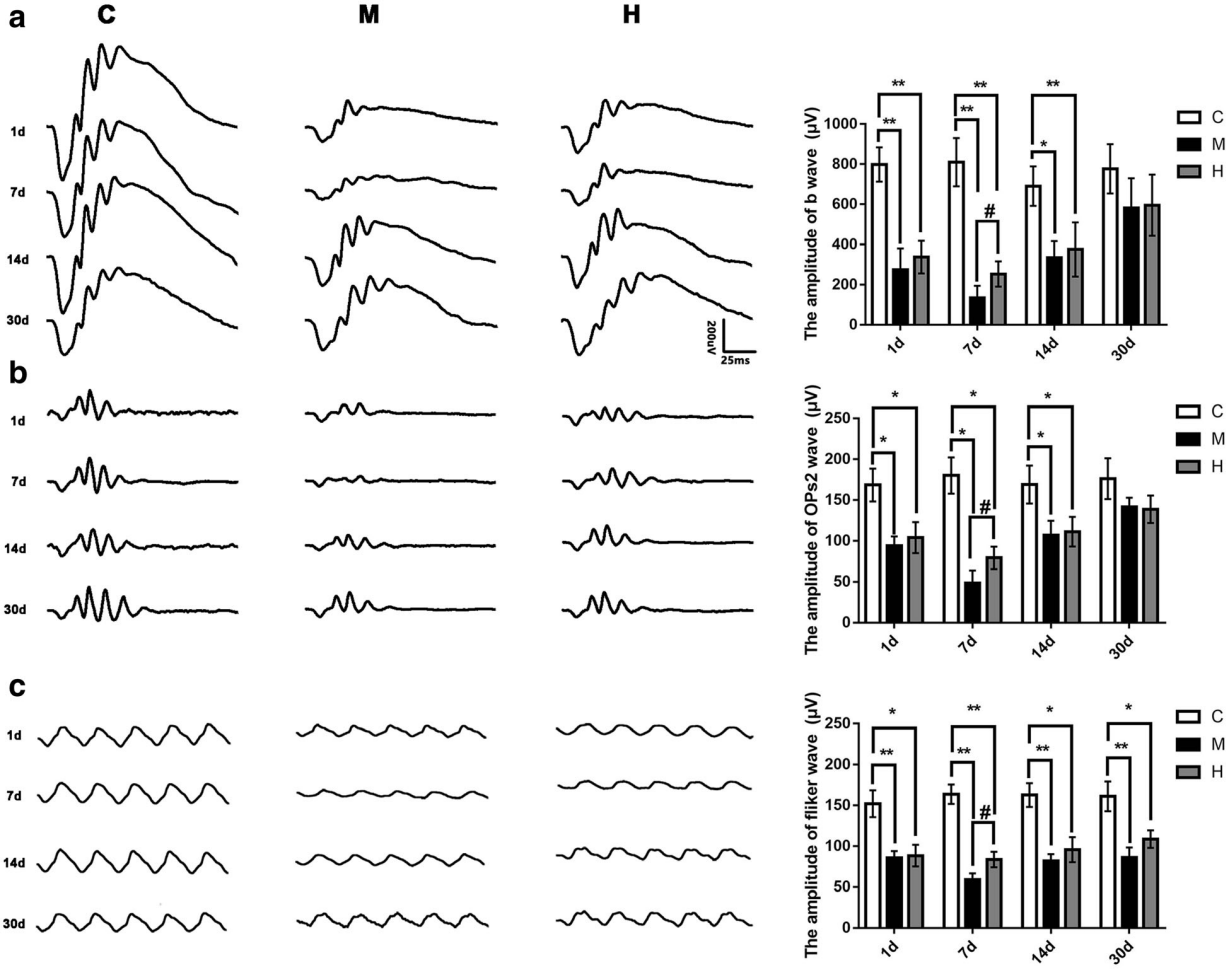
Hydrogen gas improved BRVO rat retinal function at 7 d post-occlusion. **a**: Typical dark-adaptation 3.0 response pictures and amplification of b-waves (dark-adaptation 3.0 response) at 1, 7, 14 and 30 d post-occlusion; **b**: Typical OP response pictures and amplification of OPs2 waves at 1, 7, 14 and 30 d post-occlusion; **c**: Typical light-adapted flicker response pictures and amplification of light-adapted flicker responses at 1, 7, 14 and 30 d post-occlusion. All analyses were performed in duplicate. The data are expressed as the mean ± standard deviation (SD), *n* = 4–12 rats per group. ^*^, *p* < 0.05, H and M group *vs* C group; ^**^, *p* < 0.01, H and M group *vs* C group; ^#^, *p* < 0.05, H group *vs* M group. C: control group, M: model group, H: hydrogen gas group

To evaluate the retinal function of BRVO treated with hydrogen gas, ffERG was performed. As shown in Fig. 1, the amplitudes of the b wave (dark adaptation 3.0 response) and the OPs2 wave in the H group and the M group were decreased compared with those in the C group at 1, 7 and 14 d post-occlusion (all *p* < 0.05), while there were no significant differences among the H group, M group and C group at 30 d post-occlusion (*p* > 0.05). In addition, the rats in the H group had higher amplitudes of b wave and OPs2 wave compared with those in the M group at 7 d post-occlusion (all *p* < 0.05). Interestingly, the amplitudes of the light-adapted flicker response (N-P) in the H group and M group were all dramatically decreased compared with that of the C group at every detecting point (all *p* < 0.05). Moreover, the amplitude of the light-adapted flicker response (N-P) in the H group was higher than that in the M group at 7 d post-occlusion (*p* < 0.05), and there existed no significant difference between the H group and the M group at 1, 14 and 30 d (all *p* >0.05).

### Effects of hydrogen gas on branch retinal vein recovery

**Fig. 2:**
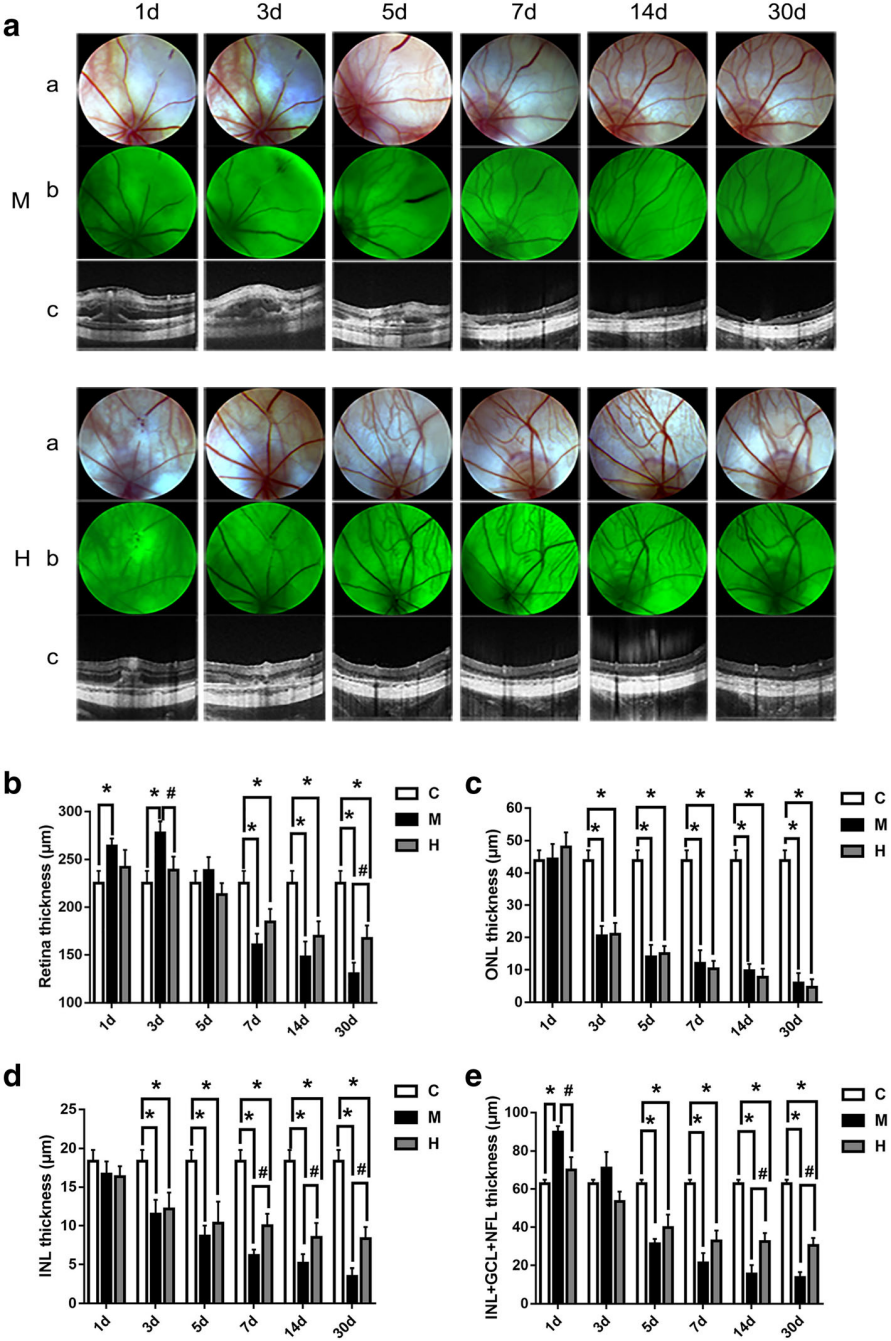
Hydrogen gas attenuated BRVO rat retinal oedema and protected the retinal structure integral. **a**: Typical fundus photograph and OCT pictures at 1, 3, 5, 7, 14 and 30 d post-occlusion; **b**: Thickness of total retina of each group at 1, 3, 5, 7, 14 and 30 d post-occlusion; **c**: Thickness of the ONL of each group at 1 d, 3 d, 5 d, 7 d, 14 d and 30 d post-occlusion; **d**: Thickness of the INL of each group at 1, 3, 5, 7, 14 and 30 d post-occlusion; **e**: Thickness of IPL + GCL + NFL of each group at 1, 3, 5, 7, 14 and 30 d post-occlusion. All analyses were performed in duplicate. The data are expressed as the mean ± standard deviation (SD), n = 4–12 rats per group. ^*^, *p* < 0.05, H and M group *vs* C group; ^#^, *p* < 0.05, H group *vs* M group. a: fundus photograph; b: fluorescent fundus colour photography. C: control group, M: model group, H: hydrogen gas group

To explore the evolution process of the occlusive vein, fundus photographs were applied at 1, 3, 5, 7, 14 and 30 d after the BRVO model was established with laser photocoagulation. The obvious occlusive vein and non-perfusion were observed in both the H group and the M group at 1 d post-occlusion. As shown in Fig. 2a, the reopen time in the H group was 2.235 ± 1.128 d, which was shorter than that in the M group (4.234 ± 2.236 d, *p* < 0.05).

### Effects of hydrogen gas on retinal structure

To evaluate the protective effect of hydrogen gas on retinal structure after BRVO in vivo, OCT was performed. For OCT, the low-reflective band contained the inner nuclear layer (INL) and outer nuclear layer (ONL), which were composed of nuclear. Moreover, the high-reflective band contained the inner plexiform layer (IPL), outer plexiform layer (OPL), photoreceptor inner segment/outer segment (IS/OS) junction line and retinal pigment epithelium (RPE) layer. As shown in Fig. 2b, OCT found that the thickness of the total retina in the BRVO area of the M group was remarkably increased for laser-induced oedema at 1 d but significantly decreased at 7, 14 and 30 d post-occlusion compared with that of the C group (all *p* < 0.05). Furthermore, there were no significant differences between the H and C groups at 1 d post-occlusion (*p* > 0.05). At the same time, the thickness of the total retina was more oedematous in the M group compared with that in the H group at 3 d post-occlusion (*p* < 0.05). Interestingly, we found that the total retina thickness in the M group was dramatically decreased compared with that in the H group at 30 d post-occlusion (*p* < 0.05). Specifically, as shown in Fig. 2c and d, the thicknesses of the ONL and INL in the H group and M group were dramatically decreased compared with those in the C group at 3, 5, 7, 14 and 30 d post-occlusion (all *p* < 0.05). Moreover, it was found that the thickness of the INL in the H group was thicker than that in the M group at 7, 14 and 30 d post-occlusion (all *p* < 0.05). As for the inner plexiform layer (IPL) + ganglion cell layer (GCL) + nerve fibre layer (NFL), as shown in Fig. 2e, those in the H group and the M group were reduced compared with those in the C group at 5, 7, 14 and 30 d post-occlusion (all *p* < 0.05). The thickness of the IPL + GCL + NFL in the M group was severely oedematous compared with those in the C group and the H group at 1 d post-occlusion (all *p* < 0.05). At 14 d and 30 d post-occlusion, the thickness of the IPL + GCL + NFL in the H group was thicker than that in the M group (all *p* < 0.05).

### Effects of hydrogen gas on microcirculation

**Fig. 3:**
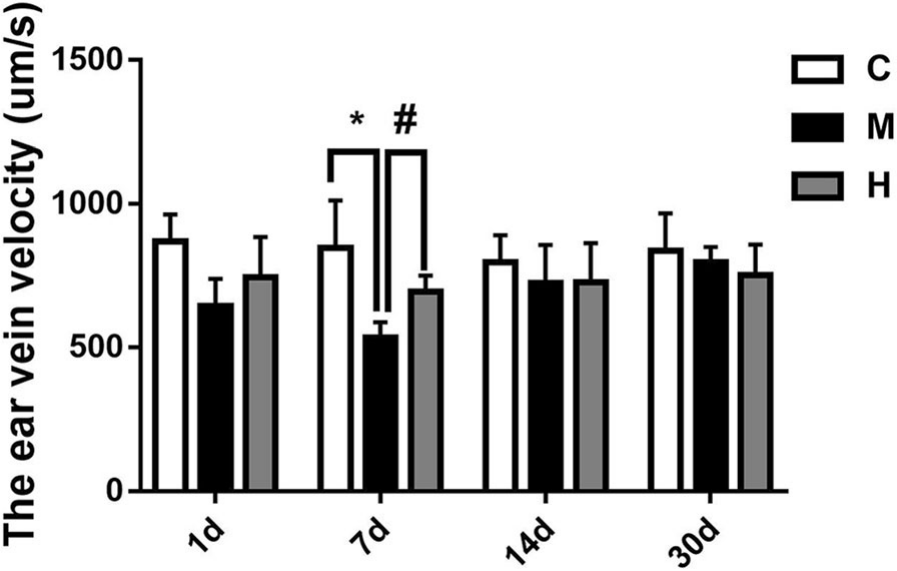
Hydrogen gas improved BRVO rat microcirculation at 7 d post-occlusion. BRVO rats ear vein blood flow velocity at 1, 7, 14 and 30 d. All analyses were performed in duplicate. The data are expressed as the mean ± standard deviation (SD), n = 4–12 rats per group. ^*^, *p* < 0.05, H and M group *vs* C group; ^#^, *p* < 0.05, H group *vs* M group. C: control group, M: model group, H: hydrogen gas group

Microcirculation was used to evaluate occlusive vein prognosis. The microcirculation detector found that the flow velocity of ear vein blood was decreased in the M group compared with that in the H and C groups at 7 d post-occlusion (all *p* < 0.05), while there was no significant difference between the H and C groups (all *p* > 0.05) (Fig. 3).

### Effects of hydrogen gas on the expression of retina VEGF-α

**Fig. 4:**
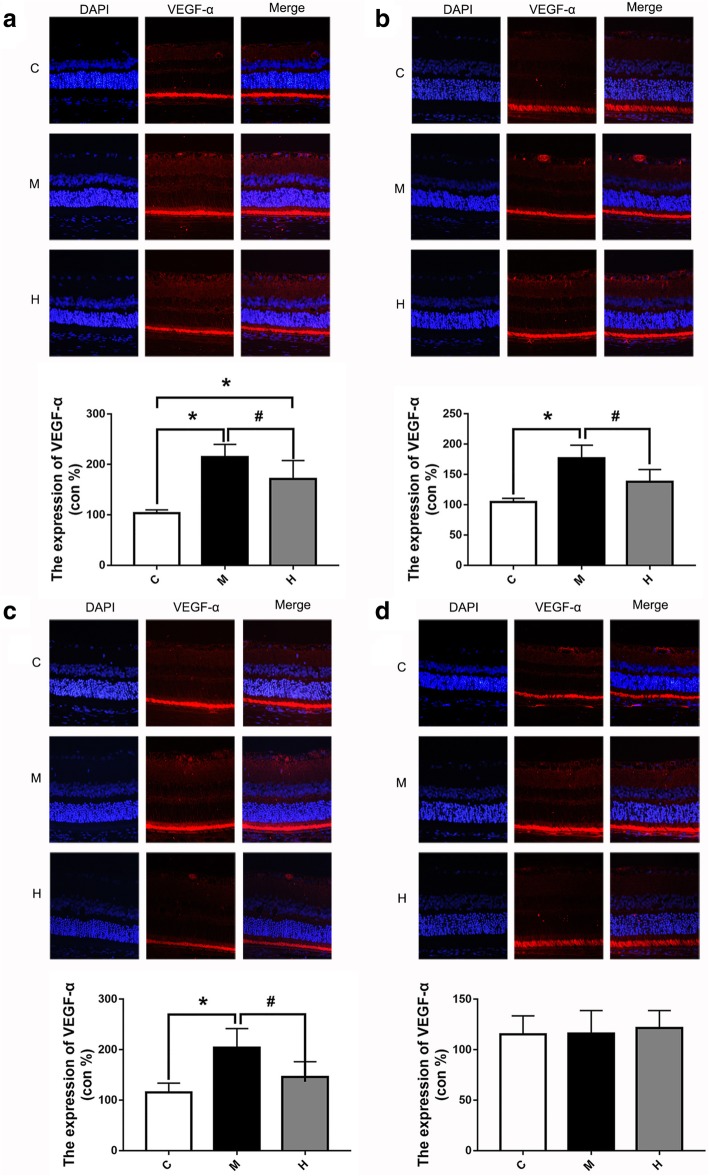
Hydrogen gas decreased the expression of VEGF-α at 1, 7 and 14 d post-occlusion. **a**: Typical immunofluorescence staining picture and expression of VEGF-α at 1 d post-occlusion. **b**: Typical immunofluorescence staining picture and expression of VEGF-α at 7 d post-occlusion. **c**: Typical immunofluorescence staining picture and expression of VEGF-α at 14 d post-occlusion. **d**: Typical immunofluorescence staining picture and expression of VEGF-α at 30 d post-occlusion. All analyses were performed in duplicate. The data are expressed as the mean ± standard deviation (SD), n = 3 rats per group. ^*^, *p* < 0.05, H and M group *vs* C group; ^#^, *p* < 0.05, H group *vs* M group. C: control group, M: model group, H: hydrogen gas group

To evaluate the expression of VEGF-α treated or untreated with hydrogen gas, immunofluorescence was applied. As shown in Fig. 4, VEGF-α was expressed mainly in the ganglion cell layer (GCL). Furthermore, the expression of VEGF-α in the H and M groups at 1 d post-occlusion was dramatically increased compared with that in the C group (all *p* < 0.05), while the expression of VEGF-α in the H group was less than that in the M group (*p* < 0.05). At 1, 7 and 14 d post-occlusion, the expression of VEGF-α in the H group was significantly lower than that in the M group (all *p* < 0.05). However, there were no significant differences among the groups at 30 d post-occlusion (all *p* > 0.05).

## Discussion

In this study, hydrogen gas via inhalation administration could protect BRVO rat retinal function recovery and retina structural integrity. We found that hydrogen gas could shorten the reopen time and improve ear vein microcirculation. Interestingly, hydrogen gas could lighten retinal oedema induced by BRVO at the early stage and prevent the retina from becoming thinner. More importantly, hydrogen gas could decrease the expression of VEGF-α to improve hypoxia in the early time of post-occlusion.

BRVO is one of the most common vessel-associated ophthalmology diseases and damages sight to different degrees [20]. It has been difficult for people to manage and treat BRVO itself due to its problematic complications. An essential reason is the lack of a suitable BRVO animal model. In the literature, people established the BRVO model by laser photocoagulation [21], diathermic cauterization, intravitreal injection of PD032590 [22], thrombin [23], NPe6 [24], or endothelin-1(ET-1) [25]. We have previously shown that modified laser photocoagulation could superbly mimic BRVO disease. In this study, the BRVO rat model was established by a modified laser photocoagulation method, which featured stabilization and homogeneity. The typical features of BRVO, such as retinal oedema, non-perfusion and reperfusion, were observed in the experiment. Retinal oedema was observed mainly at the inner retinal layer around the occlusive site at the early stage (1 d to 3 d post-occlusion). We found that retinal oedema mainly existed in the IGL, the GCL and the NFL, which was in accordance with the previous study [26]. Additionally, retinal atrophy and photoreceptor cell loss (the ONL almost disappeared) were observed at 14 and 30 d post-occlusion, which would be related to BRVO complication retinal oedema [27].

Hydrogen gas has received increasing attention in many disorders since Ohsawa demonstrated that it could remarkably suppress I/R brain injury by buffering the effects of oxidative stress. Moreover, hydrogen gas could activate the NF-E2-related factor 2 (Nrf2) cell signal pathway [13], which reduced OS by upregulating a variety of antioxidant enzymes [28]. However, the specific mechanisms of hydrogen gas have not been completely illustrated, which is urgent for further explanation. Many studies have clarified and confirmed the varying degrees of the effect of hydrogen gas, and the potential advantages have been reported: 1) hydrogen gas could rapidly diffuse across cell membranes and directly target and eliminate intracellular reactive oxygen species (ROS), such as hydroxide radical (•OH) and peroxynitrite (ONOO•); 2) hydrogen gas is a mild deoxidizer, which has little effect on the normal ROS-associated cell signal pathway [29]; and 3) hydrogen gas is obtained easily and is safe even at high concentrations [30, 31]. Considering the specific effect, hydrogen gas could easily pass through the phospholipid bilayer and be administered in BRVO to eliminate free radicals as a promising treatment.

An accurate description of the BRVO mechanism has not been totally clear. Studies have found that VEGF-α played a key role in BRVO prognosis and development [32] because it is tightly associated with BRVO complications, such as fragile neovascularization, haemorrhage and retinal oedema. Therefore, agents that target at VEGF-α have been regarded as a promising method. At present, anti-VEGF-α is a direct intervention to reduce the expression of VEGF-α, and clinical studies have confirmed that it is valid [33, 34]. However, anti-VEGF-α agents still inevitably exhibit some potential disadvantages: 1) molecular targeting drugs are expensive for BRVO patients from poor countries; 2) some patients are insensitive to anti-VEGF-α agents and even suffer severe side effects [10, 35]; and 3) VEGF-α plays an essential role in physical reactions, and anti-VEGF-α agents would influence retinal nerve cells [36–38]. It is known that excessive generation of VEGF-α is accompanied by histiocyte hypoxia, which is associated with further vascular leakage and retinal oedema [39]. However, we could not ignore the physical role of VEGF-α, which was indispensable for retina self-repair. In our study, we found that hydrogen gas inhalation could effectively decrease the expression of VEGF-α in the early BRVO stage and shorten BRVO reopen time by indirectly regulating VEGF-α. Therefore, we hypothesized that hydrogen gas could improve ischaemia reperfusion injury and could be applied to those who were insensitive to anti-VEGF-α treatment and covered with severe adverse effects. Moreover, compared with anti-VEGF-α agents, hydrogen gas could directly improve retinal hypoxia conditions, and the potential mechanisms could be related to selectively eliminating strong free radicals, inhibiting inflammatory factors and active cell survival signals [40]. In the future, we would like to explore the specific mechanisms of hydrogen gas on BRVO treatment.

Interestingly, we found that ERG function, microcirculation and the expression of VEGF-α were not significantly different in the H group compared with the M group at 30 d post-occlusion. However, we could recognize the obvious retina structural differences between the H group and the M group at 30 d post-occlusion. Moreover, we found that hydrogen gas could promote occlusive vein reopening. Meanwhile, ERG showed that hydrogen gas could reduce the rapid declines of the b-wave (dark-adaptation 3.0 response), OPs2 response and light-adapted flicker response at 7 d post-occlusion when the fundus photograph found it was close to the BRVO reopen time. We speculated that hydrogen gas could alleviate retina ischaemia-reperfusion injury, which protected retina structural integrity.

## Conclusion

The study showed that hydrogen gas played a specific role in alleviating retinal oedema, improving retinal microcirculation, protecting visual function and regulating VEGF-α expression. Taken together, the study provided curative support that hydrogen gas was beneficial for clinical application on BRVO disorder as an adjuvant treatment.

## Abbreviations

ARVO: Association for Research in Vision and Ophthalmology
BRVO: Branch retinal vein occlusion
CRVO: Central retinal vein occlusion
d3.0: dark-adaptation 3.0 response
ffERG: full-field electroretinography
Nrf2: NF-E2-related factor 2
OPs2: Oscillatory potentials
OS: Oxidative stress
ROS: Reactive oxygen species
SD: Sprague-Dawley
